# Case Report: Reactive Lymphohistiocytic Proliferation in Infant With a Novel Nonsense Variant of *IL2RG* Who Received BCG Vaccine

**DOI:** 10.3389/fped.2021.713924

**Published:** 2021-11-02

**Authors:** Amal M. Yahya, Suleiman Al-Hammadi, Nidal O. AlHashaykeh, Salwa S. Alkaabi, Abdulghani S. Elomami, Asia A. AlMulla, Majed M. Alremeithi, Rewan M. Kabbary, Ranjit Vijayan, Abdul-Kader Souid

**Affiliations:** ^1^Department of Pediatrics, Tawam Hospital, Al Ain, United Arab Emirates; ^2^College of Medicine, Mohammed Bin Rashid University of Medicine and Health Sciences, Dubai, United Arab Emirates; ^3^Department of Pediatrics, College of Medicine and Health Sciences, United Arab Emirates University, Al Ain, United Arab Emirates; ^4^Department of Pathology, Tawam Hospital, Al Ain, United Arab Emirates; ^5^Department of Hematology Oncology, Tawam Hospital, Al Ain, United Arab Emirates; ^6^Department of Biology, College of Science, United Arab Emirates University, Al Ain, United Arab Emirates

**Keywords:** SCID, IEI, *IL2RG*, BCG vaccine, Mendelian susceptibility to mycobacterial disease (MSMD), hemophagocytic lymphohistiocytosis (HLH), lymphohistiocytic proliferation, lymphoid proliferation

## Abstract

We present here a male young infant with X-linked severe combined immunodeficiency (MIM#300400) due to the novel nonsense variant of *IL2RG* (interleukin 2 receptor, gamma; MIM#308380), NM_000206.2(*IL2RG*):c.820_823dup p.Ser275Asnfs^*^29. He developed aggressive reactive lymphohistiocytic proliferation after receiving the live-attenuated Bacillus Calmette-Guérin (BCG) vaccine at birth. This report advocates for modifying the current practice of early use of BCG. The natural history of his disease also suggests considering *IL2RG* variants as a potential cause of “X-linked recessive Mendelian susceptibility to mycobacterial disease” (MSMD). His reactive lymphohistiocytic proliferation and massive hepatosplenomegaly simulated hemophagocytic lymphohistiocytosis (HLH, likely triggered by the BCG disease). This entity was masked by the absence of fever and markedly elevated inflammatory biomarkers. Thus, his findings stimulate discussion on the need to modify the diagnostic criteria of HLH, in order to accommodate conditions, such *IL2RG* variants that block systemic inflammation.

## Introduction

Bacillus Calmette-Guérin (BCG) is a live preparation derived from cultures of attenuated *Mycobacterium bovis*. Since 2005, this vaccine has been administered at birth to all newborns in the United Arab Emirates. Logically, this universal practice is expected to result in a number of “BCG disease,” especially in young infants with inborn error of immunity (IEI) or Mendelian susceptibility to mycobacterial diseases (MSMD) (1–3). An example of the latter entity is IMD28 (immunodeficiency 28, mycobacteriosis, autosomal recessive; MIM#614889), which results from pathogenic variants of *IFNGR2* (interferon-gamma receptor 2; MIM#147569), such as c.123C>G, p.Tyr41^*^ (4). X-lined examples of MSMD include pathologic variants of *CYBB*, such as p.Arg226^*^ (rs137854592), Gln231Pro (rs151344498), Thr178Pro (rs151344497), and 1662dup, p.Glu555^*^ (rs1453468510) (5). This brief report gives another example on the adverse events of BCG vaccination in a young infants with severe combined immunodeficiency due to a novel variant of *IL2RG*. It advocates for modifying its use worldwide (6–10).

## Young Infant

This 5-month-old male infant was born at term to asymptomatic, non-consanguineous emirati parents who belonged to the same tribal descent. The pregnancy and delivery were uneventful. His birth weight was 2.75 kg. He had received the BCG vaccine in the upper left arm at birth (0.05 mL, intradermal injection; manufactured by Serum Institute of India Pvt. Ltd., India). He had a healthy 16-month-old sibling, who had an oozing discharge from the BCG vaccine site for 2 months; he was currently asymptomatic. Otherwise, the family history is negative for any type of IEI.

**Figure 1:**
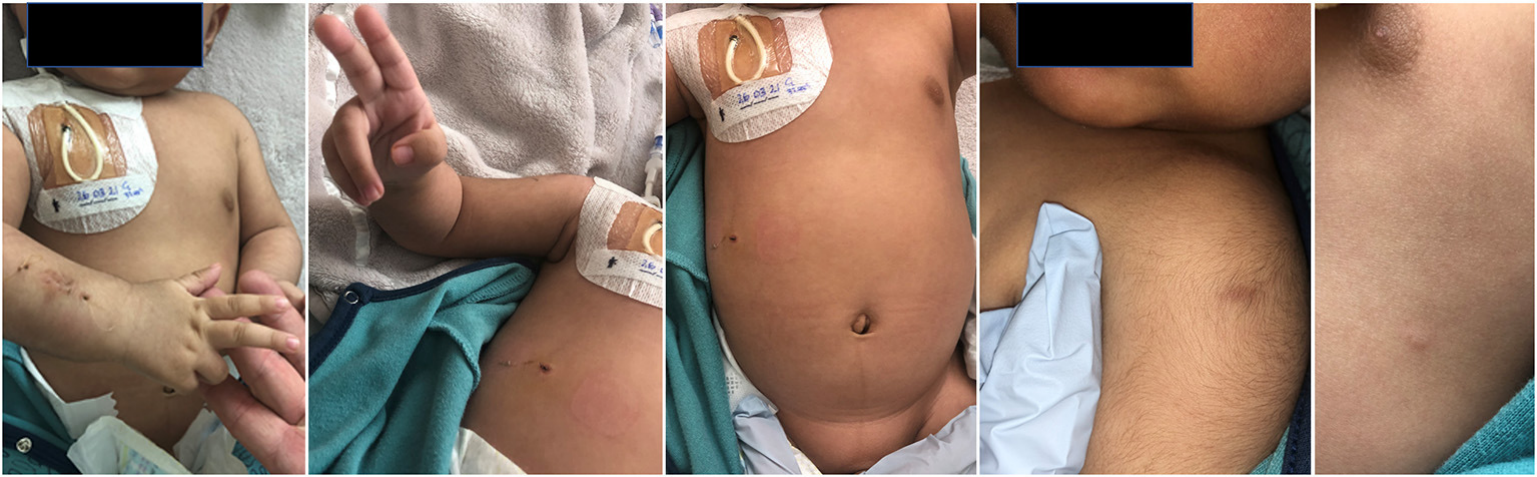
The swelling near the right wrist with the inability to move the right little and ring figures are shown. The abdominal distension (massive hepatosplenomegaly), the BCG site and a subcutaneous nodule are also evident.

His growth and development were normal. He was never genuinely febrile during the entire course of this illness; his temperatures were always ≤ 37.2°C. At 3½ months of age, he developed a tender swelling in the right lower forearm (Figure 1). Abdominal examination revealed massive hepatosplenomegaly. The skin examination was remarkable for a prominent BCG scar (Figure 1), and skin-colored soft nodules such as the one in the lateral chest cage (Figure 1). He had mild cough; but on examination, he had no shortness-of-breath and his breath sounds and oxygen saturation were normal.

His complete blood counts were remarkable for profound lymphopenia (mean ± SD lymphocyte count = 84 ± 55 per μL (median = 70; range = 20–210; *n* = 18). His serum IgA level was 0.08 g/L (normal, 0.1–0.83), IgG 1.94 g/L (normal, 2.27–13.8), IgM 0.16 g/L (normal, 0.10–1.45), and IgE 3.0 (normal, ≤ 15). Lymphocyte subset analysis was requested, but the test failed due to inadequate lymphocytes. His serum C-reactive protein (CRP) was 7.5 ± 3.5 mg/L (*n* = 8), erythrocyte sedimentation rate (ESR) 26 mm/hr, ferritin 87 ± 8 μg/L (*n* = 3), and triglyceride 1.54 ± 0.41 mmol/L (normal, 0.25–0.85). Urinalysis, coagulation profile, renal function, and hepatic function were normal. Blood cultures were negative. Bronchoalveolar lavage culture showed normal upper respiratory flora; the Grocott-Gomori's methenamine silver (GMS) stain for fungal elements was suspicious for *Pneumocystis jiroveci*, while the acid-fast bacillus (AFB) stain was negative.

**Figure 2:**
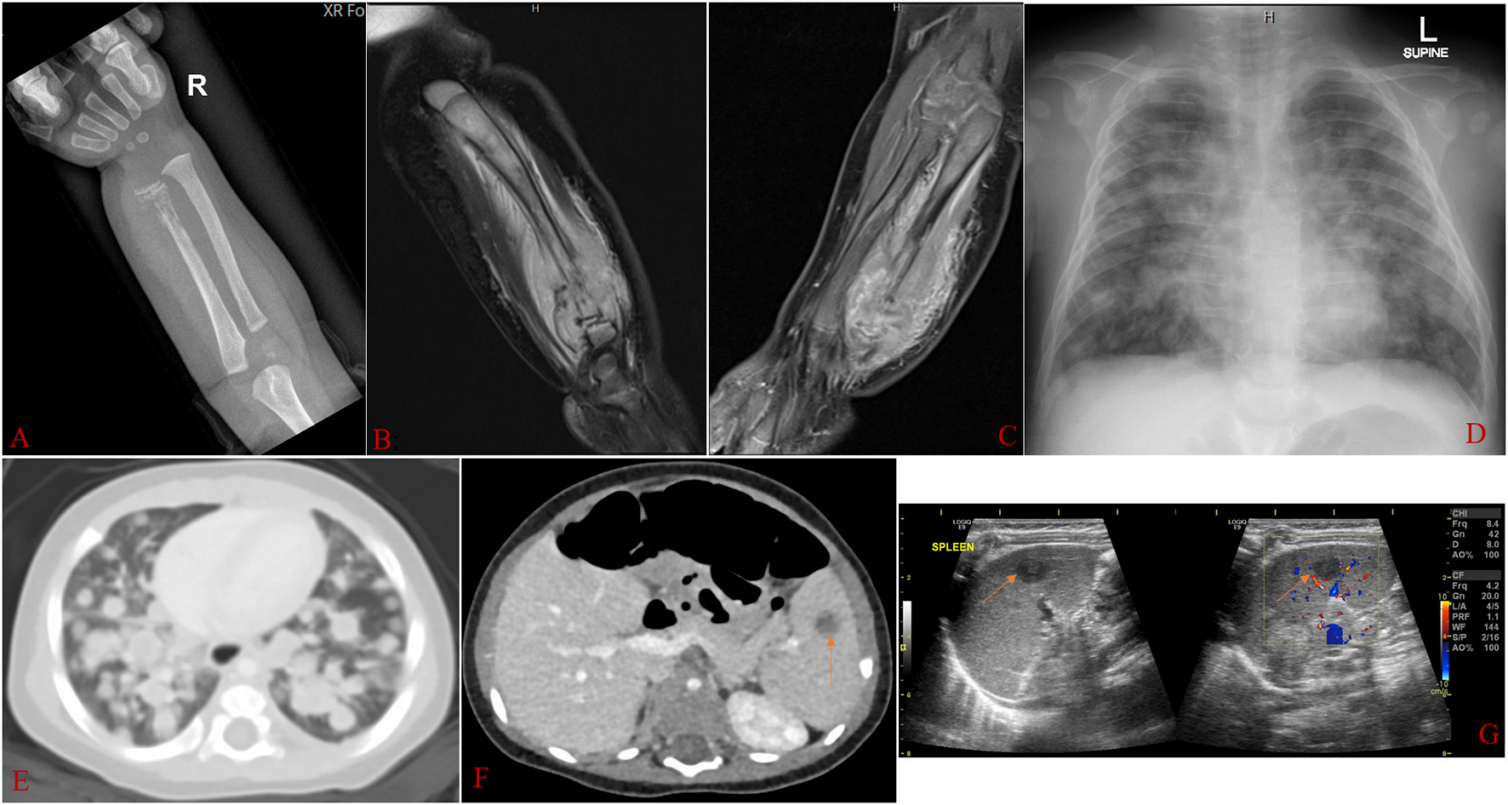
Radiographic images taken at about 3½ months of age. **(A)**: Radiograph of the right forearm showing abnormal texture in the distal ulnar, with areas of bone destruction and resorption surrounded by soft tissue swelling. **(B,C)**: MRI of the right forearm (with and without contrast) showing destructive bone lesion involving the ulnar with a large, enhancing soft tissue mass extending into the interosseous space, measuring 2.5 × 2.5 × 4.5 cm. The mass involves the diaphysis and distal metaphysis. There is periosteal reaction and edema in the surrounding soft tissue and subcutaneous fat. There is also abnormal signal and enhancement of the distal ulnar growth plate with displacement. **(D,E)**: Chest radiograph and chest CT scan showing nodular lesions in both lungs. **(F,G)**: Abdominal CT scan and ultrasound showing the right hepatic lobe exceeding the costal margin, measuring 8.6 cm craniocaudally. The spleen measures 7.0 cm in length and shows two focal hypodense lesions, the largest measuring 1.01 × 0.86 cm (arrowed).

Radiograph and magnetic resonance imaging (MRI) of the right forearm showed areas of bone destruction (resorption) in the distal ulnar, surrounded by a soft tissue mass (Figure 2). Chest radiograph and chest CT showed nodular lesions in the lungs (Figure 2). Ultrasound and computed tomography (CT) scan of the abdomen showed organomegaly with two focal hypodense splenic lesions (Figure 2).

**Figure 3:**
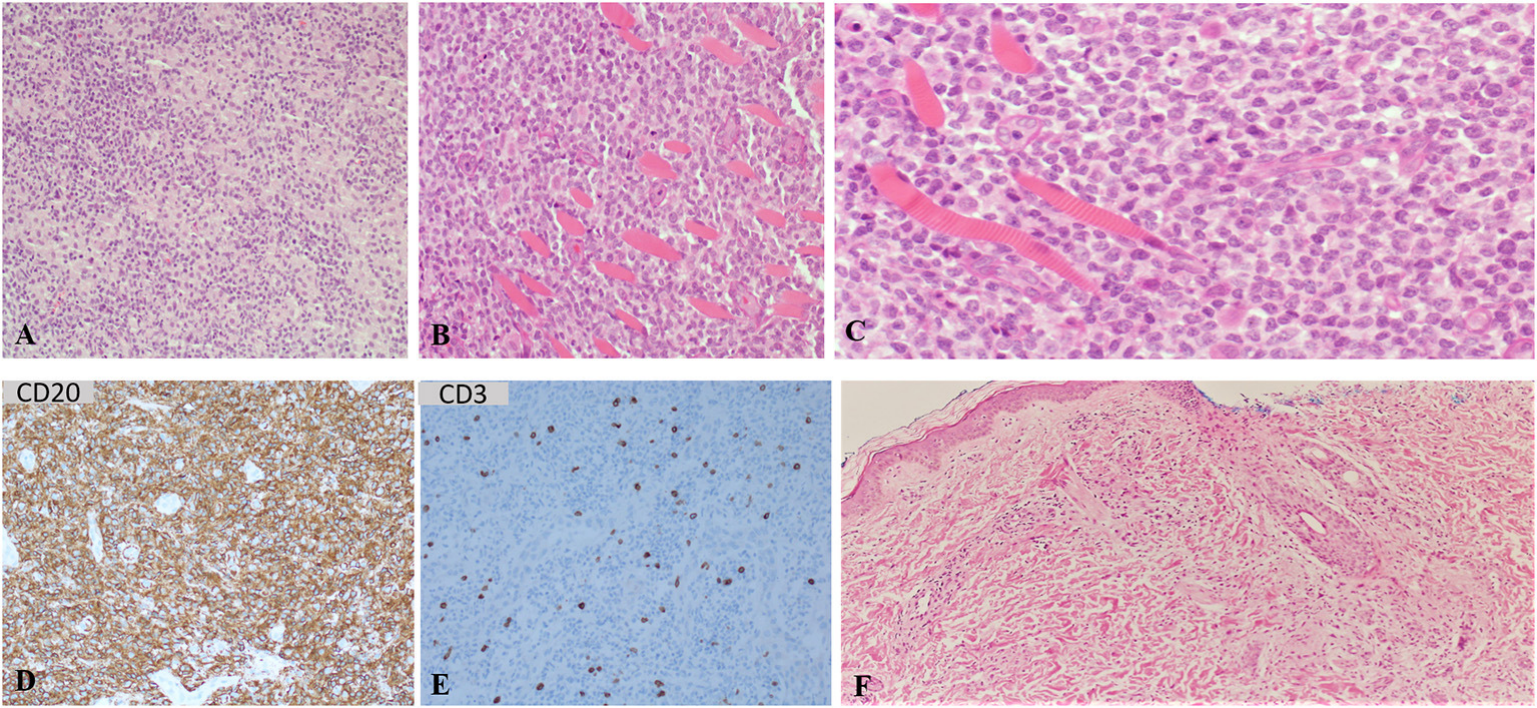
Sections of routine stain from the soft tissue mass right distal ulna. **(A)** shows sheet of mononuclear cells of lymphocytes and histiocytes. **(B)** shows predominate lymphocytic infiltrate bone and skeletal muscle. **(C)** shows higher magnification of the right ulnar lesion routine stain section shows mononuclear cells predominantly of lymphocytes with scattered mitosis infiltrating skeletal muscle (skeletal muscle appears as eosinophilic striated tissue with peripheral nuclei at left side of the image). Most of the lymphocytes are B cell with CD20 positive immunohistochemical stain **(D)**, mixed with few scattered T lymphocytes highlighted with CD3 immunohistochemical stain **(E)**. Skin biopsy shows dermal inflammatory cells infiltrate, mainly of histiocytes with granulomatous inflammation **(F)**.

Biopsies of the right forearm soft tissue mass and the ulnar bone lesion revealed “*reactive lymphohistiocytic proliferation*,” as a part of infection such as the administered live vaccine BCG. Morphologically, the specimens showed infiltrating lymphohistiocytic proliferations by small-to-medium size mature/activated B cells [PD1 (programmed cell death protein 1) positive] of non-germinal center origin intermixed with normal histiocytes (Figures 3A–E). For both specimens, fluorescence *in-situ* hybridization (FISH) panel for B cell lymphoma [detects rearrangements involving *IGH* (immunoglobulin heavy chain; MIM#146910), *C-MYC* (MYC protooncogene, bHLH transcription factor; MIM#190080), *BCL2* (B-cell lymphoma 2; MIM#603167), *BCL6* (B-cell lymphoma 6; MIM#109565), *CCND1* (cyclin D1; MIM#168461), and *MALT1* (mucosa-associated lymphoid tissue lymphoma translocation gene 1 paracaspase; MIM#604860)] was negative. Immunohistochemical stains of CD10, BCL6, CD30, C-MYC, CD34, TdT, myeloperoxidase, Cyclin D1, AE1/AE3 (bind to cytokeratins and serve as a marker of carcinomas), S100 proteins, CD99, myogenin, desmin, and MyoD1 were also negative. The Ki-67 stain was positive in about 50% of the cells. *In-situ* hybridization with EBV encoded mRNA was negative.

Biopsy of a skin nodule revealed dermal histiocytic infiltrate forming granuloma (Figure 3F).

Acid-fast bacillus (AFB) stain was negative for *mycobacterium tuberculosis*, and Grocott-Gomori's methenamine silver (GMS) stain was negative for fungal elements. Bone marrow biopsy showed cellular marrow (>90%) with trilineage hematopoiesis, adequate maturation, and T cell depletion.

Diagnostic exome sequencing revealed the hemizygous, pathogenic, novel non-sense variant of exon 6 of *IL2RG* (interleukin 2 receptor, gamma; MIM#308380): NM_ 000206.2(*IL2RG*):c.820_823dup, p.Ser275Asnfs^*^29. This novel variant causes “severe combined immunodeficiency, X-linked” (SCIDX1 or XSCID; MIM#300400). This *IL2RG* variant was not detected in the mother's blood sample. Thus, the variant was assumed *de novo* in the affected infant. Germline mosaicism, however, could not be excluded. It is also important to note that the only significant variant reported on his diagnostic exome sequencing test was that involving the *IL2RG* gene. In addition, there was no clinically relevant copy number variant by NGS-CNV (copy number variation detection by next generation sequencing read counts) analysis.

His management included intravenous immunoglobulin, sulfamethoxazole-trimethoprim, isoniazid, rifampin, vancomycin, piperacillin/tazobactam, azithromycin, and amphotericin B liposomal. He continued breastfeeding and occasionally required oxygen supplementation by nasal cannula. He had no human leukocyte antigen (HLA) identical match. Thus, he was transferred to a transplant center for parental donor haploidentical hematopoietic stem cell transplantation (HSCT).

## Discussion

This male infant had SCID (MIM#300400) due to the novel non-sense variant, NM_000206.2(*IL2RG*):c.820_823dup p.Ser275Asnfs^*^29 (11). He developed an aggressive reactive lymphohistiocytic proliferation after the BCG vaccine (4). Evidenced by the skin nodule histology, this illness is most likely a BCG disease as a result of his inborn error of immunity (IEI). It is well to know that sensitivities of the special stains acid-fast bacillus (AFB) and Grocott-Gomori's methenamine silver (GMS) are low. Therefore, false negative stains in situations of infection are relatively common. In a recent review, the sensitivity of “AFB microscopy” ranges from 20 to 70%, with a specificity of 95% or higher (12). In one study, the sensitivity of GMS stain for invasive pulmonary aspergillosis was 92% and specificity 82%; the negative predictive value was 75%, and the positive predictive value was 95% (13).

The clinical and pathologic data in this infant confer a predisposition to the weakly virulent mycobacteria, such as that of the BCG vaccine (4). Thus, this monogenic inherited condition provides an additional insight into the known list of MSMD (Mendelian susceptibility to mycobacterial diseases) (1–3). This report also advocates for modifying the current practice of early use of BCG (8–10).

The natural history of his disease suggests considering *IL2RG* variants as a potential cause of “X-linked recessive Mendelian susceptibility to mycobacterial disease.” His reactive lymphohistiocytic proliferation and hepatosplenomegaly simulated hemophagocytic lymphohistiocytosis (HLH), likely triggered by the BCG disease. The lack of fever and the just mildly elevated inflammatory biomarkers (serum ferritin, triglyceride, CRP, and ESR) are likely explained by the defective “interleukin 2 receptor, gamma” (14).

**Figure 4:**
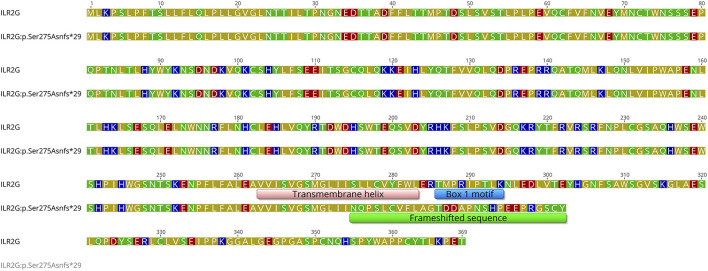
A sequence alignment of the wild-type ILR2G and the variant ILR2G: p.Ser275Asnfs*29 proteins. The transmembrane region and the “Box 1 motif” regions of the wild-type protein are annotated along with the frameshifted sequence of the variant ILR2G. Amino acids are colored based on their polarity: blue - basic; red - acidic; green - polar; olive - hydrophobic.

There are no *in silico* pathogenicity predictions for this novel variant (p.Ser275Asnfs^*^29) from Ensembl Variant Effect Predictor (VEP) (15), but Varsome (16) indicates that it is pathogenic. The frameshift variation results in the alternation of the polypeptide sequence starting from position 275. This position is located in the middle of the single transmembrane helix (amino acids 263-283) of the IL2RG cytokine receptor. The 4-base duplication (c.820_823dup) results in a frameshift and the truncation of the polypeptide 29 amino acids downstream. Transmembrane helix prediction using TOPCONS (https://topcons.cbr.su.se) indicates that the variant peptide could still harbor a transmembrane helix in this region. Critically, the region 286-294, termed the “Box 1 motif,” is required for the activation of Janus kinase (JAK), a tyrosine kinase that links cytokine signaling to transcription factors, by ILR2G (14). The consensus sequence of the “Box 1 motif” includes several hydrophobic residues and conserved prolines. The duplication significantly alters the protein sequence between 275 and 302; including the intracellular “Box 1 motif,” and truncates the polypeptide at position 303 (Figure 4). The “Box 1 motif” region lacks the necessary hydrophobic residues and the conserved prolines and, therefore, the variant protein is unlikely to activate JAK.

Interleukin 2 receptor gamma (IL2RG) is present in the receptors for IL-2, IL-4, IL-7, IL-9, IL-15, and IL-21 (17–20). Stimulation of these receptors causes specific tyrosine phosphorylation and activation of JAK1 and JAK3. JAK3 is selectively associated with the c-terminal region of IL2RG, which is vital for the thymic development of T cells (14). Thus, the profound T cell lymphopenia in this infant with *IL2RG*:p.Ser275Asnfs^*^29 is a result of the failure of IL2RG-JAK3 complex to promote thymic T cell selection and maturation (21, 22).

## Data Availability Statement

The original contributions presented in the study are included in the article/supplementary material, further inquiries can be directed to the corresponding author/s.

## Ethics Statement

The studies involving human participants were reviewed and approved by Tawam Human Research Ethics Committee - Signed informed consent was obtained from the parents. Written informed consent to participate in this study was provided by the participants' legal guardian/next of kin. Written informed consent was obtained from the individual(s) for the publication of any potentially identifiable images or data included in this article.

## Author Contributions

A-KS, AY, and SA-H: conceived, designed, structured the report, edited, and reviewed the paper. NA, SA, AY, AA, MA, and RK: analyzed and interpreted the clinical data. AE: analyzed and interpreted the pathology data. RV: analyzed and interpreted the variant. A-KS, AY, AE, and RV: wrote the first draft. All authors contributed to the article and approved the submitted version.

## Conflict of Interest

The authors declare that the research was conducted in the absence of any commercial or financial relationships that could be construed as a potential conflict of interest.

## Publisher's Note

All claims expressed in this article are solely those of the authors and do not necessarily represent those of their affiliated organizations, or those of the publisher, the editors and the reviewers. Any product that may be evaluated in this article, or claim that may be made by its manufacturer, is not guaranteed or endorsed by the publisher.
